# Centenary of Karl Jaspers’s general psychopathology: implications for molecular psychiatry

**DOI:** 10.1186/2049-9256-2-3

**Published:** 2014-05-16

**Authors:** Johannes Thome

**Affiliations:** Department of Psychiatry, University of Rostock, Gehlsheimerstraße 20, 18147 Rostock, Germany

**Keywords:** Brain, Methodology, Mind, Neuroscience, Philosophy, Materialism, Reductionism, Theory of science

## Abstract

Modern molecular psychiatry benefits immensely from the scientific and technological advances of general neuroscience (including genetics, epigenetics, and proteomics). This “progress” of molecular psychiatry, however, will be to a degree “unbalanced” and “epiphytic” should the development of the corresponding theoretical frameworks and conceptualization tools that allow contextualization of the individual neuroscientific findings within the specific perspective of mental health care issues be neglected. The *General Psychopathology*, published by Karl Jaspers in 1913, is considered a groundbreaking work in psychiatric literature, having established psychopathology as a space of critical methodological self-reflection, and delineating a scientific methodology specific to psychiatry. With the advance of neurobiology and molecular neuroscience and its adoption in psychiatric research, however, a growing alienation between current research-oriented neuropsychiatry and the classical psychopathological literature is evident. Further, consensus-based international classification criteria, although useful for providing an internationally accepted system of reliable psychiatric diagnostic categories, further contribute to a neglect of genuinely autonomous thought on psychopathology. Nevertheless, many of the unsolved theoretical problems of psychiatry, including those in the areas of nosology, anthropology, ethics, epistemology and methodology, might be fruitfully addressed by a re-examination of classic texts, such as Jaspers’s *General Psychopathology*, and their further development and adaptation for 21st century psychiatry.

## Review

### Introduction

The young Karl Jaspers (30 years of age) published the first edition of his *General Psychopathology* at a time when psychiatry had only recently been solidly established as a medical and scientific discipline, and ambitious research projects, such as that of Kraepelin, were inaugurated in order to uncover the fundamental bases of psychiatric conditions. The enthusiasm for purely biological approaches in psychiatry according to Griesinger’s dictum that all psychiatric disorders were brain disorders, however, was already meeting with increasing skepticism; Freud, for example, who had commenced his career as a neuropathologist, had already decided to proceed within a totally different theoretical framework, a psychological approach he termed psychoanalysis. Jaspers seemed to strongly feel the need to critically evaluate psychiatric methodology, and to develop a methodological system that would be based on “dynamic” principles, permitting continuous re-adjustment as the discipline of psychiatry generated increasing knowledge about the mind and the brain [1].

In parallel with these genuine scientific-philosophical considerations, Jaspers also composed his work in order to foster his academic career. It was submitted to the University of Heidelberg as his “habilitation” thesis, in order to obtain his license to teach psychology at the university; it also enabled him to move from the Medical Faculty to the Faculty of Philosophy. This practical aspect shares some similarities with the preparation of the *Tractatus logico-philosophicus* by Ludwig Wittgenstein, who similarly employed his labors to the advantage not only of philosophy, but also in order to earn his doctoral degree from Cambridge University. An acquaintance with the biography of Karl Jaspers will greatly enhance the ability to analyze and comprehend his work and to place it in its historical context.

### Karl Jaspers: biography

Born in 1883 in Oldenburg (Germany), Karl Jaspers suffered poor health throughout his youth (hereditary bronchiectasis) which, according to his own testimony, shaped his character to a certain extent, as it rendered him physically fragile and thus required stringent discipline in order to overcome his weakness. Born into an upper middle-class family, he was able to study law for a few semesters in Heidelberg and Munich before beginning his medical training during 1902 in Berlin, Göttingen and Heidelberg. In 1908, he obtained his medical degree, and worked until 1914 as a junior physician with Franz Nissl, who was at this time Director of the University Psychiatric Hospital in Heidelberg [2]. With the publication of the *General Psychopathology* he began a career as philosopher, and never returned to practicing medicine or psychiatry. Instead, he became, together with Martin Heidegger, one of the most important representatives of German existentialism [3]. Unlike Heidegger, however, he opposed National Socialism from the outset; further, he refused to divorce his Jewish wife, as a result of which the couple lived in constant threat. Jaspers was forced by the regime into premature retirement from the university in 1937; a year later he was also barred from publishing.

From 1945 to 1948, he actively supported the re-establishment of free academic life at Heidelberg University and other academic centers. He was disappointed by political developments in post-war Germany, however, and emigrated to Switzerland in 1948, where he obtained a professorship in philosophy at Basle University. He adopted Swiss citizenship, and died in Basle in 1969.

### The book

The *General Pathology* has appeared in no less than eight editions since its first publication in 1913 [4] to the latest edition immediately before its author’s death. Jaspers contributed a significant amount of fresh material to each new edition, with the consequence that the eighth edition (1965) was significantly more voluminous than the first. Later editions also contain interesting comments by Jaspers regarding the reception and misperceptions of his work. Successive editions naturally differ considerably from the first, so that detailed analysis also needs to reflect the metamorphosis of this work over time. The fourth edition, in particular, represents a fundamental revision of the earlier publications [3]. This paper will be based on the fifth edition of 1948 [5], together with consideration of the first in order to clearly distinguish between the original work and later additions.

While the *General Psychopathology* is generally considered one of the major works in psychiatric literature, it is interesting to notice that very few papers published over the past 20 years in Medline-listed psychiatric, medical and scientific journals have concerned themselves with his thought (0–3 articles per year). In the centenary year of 2013, on the other hand, around 25 articles can be retrieved if one searches for titles including “Jaspers” and “psychopathology”. This is clearly an indication that Jaspers’ work is remembered for historical reasons, but seems to play only a minor role in current scientific discourse in psychiatry. With the advance of molecular psychiatry, the reception and reflection of Jaspers’s psychopathology seems to be receding ever further.

### Molecular psychiatry

While the so-called biological psychiatry of the 1970s, 1980s and 1990s was mostly based upon quite reductionist (and retrospectively simplistic) hypotheses about psychiatric conditions (such as simple neurotransmitter hypotheses developed in analogy to models of neurological disorders, such as Parkinson’s disease), the introduction of more advanced molecular neuroscience methods [6, 7] makes it possible to apply hypothesis-generating approaches in modern molecular psychiatry, and also to integrate genetic aspects into environmental models (including social and psychological aspects) of psychiatric conditions [8]. Blinkered reductionist thought has given way to a much broader approach that allows space for questions about philosophical aspects of psychiatry, including its methodology, anthropology, and ethics [9]. From a therapeutic perspective, the focus is no longer purely on biological (for example, pharmacological) or psychotherapeutic approaches, but on an integration of both, with the goal of a unique and individualized therapy for each patient. Most practicing psychiatrists would probably describe themselves as non-reductionist materialists who are not only willing but keen to critically reflect upon their actions in clinical practice and research. However, this discourse requires a non-biological terminology and methodology that could at least partially be provided by Jaspers’s *General Psychopathology*.

### Differences between the philosophical/psychopathological approach and modern psychiatry/neuroscience

Integrating psychopathological thinking into modern molecular psychiatry might at least initially be difficult due to a number of significant differences. Formally, discoveries in molecular psychiatry are published in relatively short journal articles with a very short “half-life” of sometimes only a few months. In contrast, psychopathological thinking is – similar to philosophical thought – outlined in rather voluminous books whose content can remain relevant for more than a hundred years (such as Jaspers). Further, classic psychopathological work is not always published in the current lingua franca (English), and translations of appropriate quality are not always available, complicating the reception of such work.

Methodologically, the philosophical method of developing ideas and arguments based upon logic, conjectures and refutations, as applied to psychopathology, is quite different from the empirical-experimental approach employed in molecular psychiatry [10]. A tendency towards reductionism (required in order to establish experimental settings) in the latter contrasts with an attempt by the former to understand “the big picture” in its entirety.

There nevertheless exists a common basis for trying to better understand the mind and brain, and to fundamentally comprehend practical psychiatry. In this context, Jaspers often used the word “elucidation” (*Erhellung*), a term often found in modern scientific texts.

### Psychopathology as methodology

Jaspers underscored the contrast between an individual patient with their personal history, and the experience of complex states or conditions that are independent of individuals. He postulated that the expression (*Ausdruck*) and facts (*Tatbestände*) of this condition need to be differentiated, and that these terms must not be confused with the mental (*das Psychische*), which cannot be investigated *per se*.

He also discussed the relation between general psychopathology and somatic medicine, and spoke of a deep unity (*innige Einheit*) between the mind/psyche and the physical/somatic. Nonetheless, a 1:1 correspondence between the two is not possible, despite certain “parallelisms” and interdependencies. It rather is like a “continent observed from two sides”, an “ocean whose coastline is examined”. He therefore postulated that general psychopathology should be freed from the slavery (*Knechtschaft*) imposed by the dogma that psychiatric disorders are brain disorders.

Nevertheless, psychopathology is, according to Jaspers, intimately related to medicine and psychology. If the potential of psychopathology is denied, and progress identified only with “histology and serology”, then there exists a confusion between the progress represented by discoveries of physical phenomena and the autonomous progress of psychopathology, which for Jaspers is defined by its methodology.

### Terminology

Jaspers attempted to derive a specific psychopathological terminology by discussing three central problem areas: comparison of human beings and animals in order to define their similarities and dissimilarities; objectification (and organization) of the soul; and the interaction between the inner and outer worlds of the individual. The first area provided a useful somatic terminology because of identified somatic similarities. The dissimilarities lead to terms such as “personhood” (*Menschsein*), “mind” (*Geist*), and “human soul” (*Menschenseele*), and also give rise to the question whether animals can suffer from mental illnesses, which would be necessary, were animal models in psychiatry to be valid; this remains a highly contested question in current empirical molecular-psychiatric research. It also reminds one of Tim Crow’s dictum that “schizophrenia is the price that *homo sapiens* pays for language” [11], and other unique features of being human.

Jaspers also stressed that the soul *per se* is not the direct object of the psychopathological project. The soul becomes rather indirectly an object via phenomena that can be perceived, that is, via matters of fact (*Tatbestände*), including somatic (organic) data. The soul/mind itself remains the all-encompassing that cannot be objectified itself. This train of thought on the soul/mind (*Seele*) provided key psychopathological terms, such as consciousness (inwardness of experience: *Innerlichkeit derErfahrung*), representational consciousness (*objektivesBewusstsein*), self reflection (*Selbstreflexion*), existence in one's world (*Sein in ihrer Welt*), as well as becoming (*Werden*), development (*Entfaltung*), and differentiation (*Differenzierung*), terms which also signify its non-finality (*nicht engültig*) and incompletion (*nicht vollendet*).

Finally, the interaction between the inner world (*Innenwelt*) and the environment (*Umwelt*) makes it necessary to interpret “life” from a psychopathological view as existence in its world (*das Leben als Dasein in seiner Welt*), which again unlocks a thesaurus of terms that have, in part, become part of current molecular psychiatry (gene-environment interaction, stimulus-reaction, ego-subject matter, subject-object, predisposition-milieu).

### Prejudices

Jaspers listed several prejudices with which psychopathology can be confronted and which need clarification:

The philosophical prejudice or misconception assumes that psychopathology employs merely deductive methods; that is, it derives conclusions from a hermetic set of preconceived theoretical ideas, whereas psychopathology, in fact, is based upon a pro-science (pro-empirical) outlook. So-called neurophilosophy had, however, not yet been developed at the time when Jaspers was writing; this form of “empirical philosophy” aims at unifying theories of the mind and the brain [12] according to a radically scientific and neurobiological approach which assumes that even philosophy can be replaced by neuroscience, a view that has often been criticized as being overly naïve and reductionist.

The theoretical prejudice sees psychopathology as a “unifying theory”, but Jaspers actually argued that the mental world (*Seelenleben*) cannot be reduced (*zurückgeführt werden*) to a few universal principles. It would therefore be wrong to metaphorically compare the soul/mind to an ocean, with psychopathology discovering and describing more and more of its shores and islands.

The somatic prejudice identifies psychopathology with physiological, anatomical and/or general biological phenomena and processes. In this context, Jaspers states that identifying the methodology underlying Meynert’s and Wernicke’s work on aphasias and apraxias with genuine psychopathological practice would lead to “brain mythology” (*Hirnmythologie*) [13]. Nevertheless, Jaspers underscored repeatedly the fact that the psychological and the somatic (*das Seelische und das Körperliche*) are intertwined through parallelisms and interactions (*Wechselwirkungen*).

The psychological-intellectualistic prejudice implies an interpretation of psychopathology that considers it as pure speculation without any basis in reality. Such a stance, however, has rendered it impossible for so-called ‘somaticists’ to accept phenomena such as hysteria.

The image prejudice is defined by a reduction of psychopathological phenomena to pure images (such as spatial images) that are misinterpreted as the actual design of the soul/mind (*Bilder als “Seelengrundrisse”*).

The medical prejudice, finally, challenges any psychopathological conceptualization by postulating that any such project must be based solely upon quantifiable phenomena. By pointing out that psychopathology must go beyond purely measurable parameters, Jaspers has anticipated the raise of so-called qualitative research methods, which are today attracting increasing attention, and largely derive their theoretical framework from French postmodernist philosophy (including Lacan, Foucault, Derrida).

### Prerequisites

After delineating possible routes to the required psychopathological vocabulary and discussing possible stumbling blocks to the project (prejudices), Jaspers discussed the necessary prerequisites for successful engagement in psychopathology. Firstly, it is necessary to be thoroughly competent in its specific methodology and its application (*Beherrschen der Methode*). Secondly, openness and the ability to see and experience are required (*Offenheit, Seh- und Erlebensfähigkeit*). Thirdly, all prejudices must be eliminated. Finally, one must possess the capacity for self-criticism (*Selbstkritik*).

### Methods

Psychopathological methodology is divided by Jaspers into “technical” and “logical” aspects. The technical methods consist of casuistics (in contrast to the practice of an ever-increasing number of modern psychiatric journals, whose editors consider case reports as unworthy of publication), statistics, and experiments.

The logical methods include the apprehension of individual phenomena (*Auffassung von Einzeltatsachen*), that is, phenomenology; the examination of contexts and relations (*Erforschung von Zusammenhängen*), leading Jaspers to his well-known differentiation between understanding (*verstehen*; that is, examination of the subjective inner world) and explaining (*erklären*, examination of the objective outer world, and thereby the main focus of so-called biological psychiatry); and the “embracement of the whole” (*Ergreifen der Ganzheit*).

### Errors

By discussing possible errors which might lead psychopathological (and other) research astray (*Abwege*), Jaspers anticipated several problems within the theoretical framework of current psychiatry that have still not been solved:

According to Jaspers, the key problems are related to being overpowered by boundlessness (*Überwältigungdurch die Endlosigkeit*). In this context he cited the infinitude of case histories, the endless enumeration of the countable (*Zählbares zählen*), the infinitude of correlations and of elements, combinations and permutations. He further mentioned the infinitude of “auxiliary constructions” (*Hilfskonstruktionen*) and of “potentialities” (*Endlosigkeit des Allesmöglichen*). He further criticized the risk of “literary endlessness” (*literarische Endlosigkeit*), thereby anticipating the modern madness of “publish or perish”.

Two further errors to be avoided were “becoming bogged down by dogmatism” (*Festfahren in der Verabsolutierung*), that is, the risk of doctrinism that is especially virulent in academic circles where psychopathology is taught as a “school of thought” (such as the Leonhard school [14], the Heidelberg school etc.); and sham insights through inappropriate use of terminology (*Scheineinsicht durch Terminologie*), a problem that haunts commissions established to edit consensus-based “classification criteria”, the ever increasing number of new editions of which represents little more than faux progress and a deceptive advance of psychiatry.

## Conclusion

For Jaspers, psychopathology consisted of methodology and methodological criticism. He defined methodology as the combination of methodological consciousness and methodological system (*Ordnung*), as the principle of classification (*Gliederung*), and as isolation of the single methods as well as the whole picture (*Gesamtbild*). This is the central narrative of his work. It is accordingly a misunderstanding to identify his thought with phenomenology, biology or the difference between them (as has often been in the course of the reception of his work). Like early Socratic dialogues, the *General Psychopathology* appears to end aporetically regarding the issue of how firm results can be obtained within this definition of psychopathology. Jaspers, however, believes that at least an approach to (*Annäherung*) and an encircling (*Einkreisen*) of universal “truth” is, at least in principle, possible [15]. This “hermeneutic circle” also requires acceptance of open questions regarding knowledge (*offene Erkenntnisfragen*) and an awareness of one’s own limits (*Grenzen*). The experience of limits or border situations (*Grenzerfahrung*) is a central term of Jaspers’s philosophy [16], and therefore also of his psychopathology.

Jaspers respected the achievement of the natural sciences, but rejected empty verbal formula (as are sometimes peddled in the humanities). Natural sciences are for him not an alternative to psychopathology, but an integral component of it.

Psychopathology as defined by Jaspers is thereby a chance for molecular psychiatry to look beyond its own biologistic borders and to overcome its solipsism, frustration, and lack of orientation. Psychopathology can assist finding answers to essential questions that cannot be addressed by neurobiological means alone, but are fundamental to psychiatry.
